# Growing Oriented Layers of Bi_4_Ti_3_O_12_ in Bi_2_O_3_/TiO_2_/SiO_2_/Nd_2_O_3_/Al_2_O_3_ Glass-Ceramics by Melt Quenching

**DOI:** 10.1038/s41598-018-26876-6

**Published:** 2018-06-05

**Authors:** Wolfgang Wisniewski, Stanislav Slavov, Christian Rüssel, Yanko Dimitriev

**Affiliations:** 1 Otto-Schott-Institut, Jena University, Fraunhoferstr. 6, 07743 Jena, Germany; 2 University of Chemical Technology and Metallurgy, Department of Physics, 8 Kl. Ohridski Blvd, 1756 Sofia, Bulgaria

## Abstract

A glass melt with the composition 24Bi_2_O_3_/40TiO_2_/10SiO_2_/10Nd_2_O_3_/16 Al_2_O_3_ was prepared and rapidly quenched between two copper blocks (sample A). A part of this glass was subsequently crystallised at 800 °C for 8 h (sample B). For the preparation of another two samples, the melt was slowly cooled on a cooper plate (sample C) or cast into a graphite mould and subsequently thermally treated at 300 °C for 3 h (sample D). As shown by X-ray diffraction (XRD) and scanning electron microscopy (SEM) including energy dispersive X-ray spectroscopy (EDXS) and electron backscatter diffraction (EBSD) measurements, the rapidly cooled samples contained notable amounts of uncrystallised glassy phase next to the Aurivillius phase Bi_4_Ti_3_O_12_. The latter occurred in higher concentrations in all other samples and formed oriented layers. Minor concentrations of Bi_2_Al_4_O_9_ and Al_2_O_3_ were also detected in the microstructure.

## Introduction

The excellent ferroelectric properties of crystalline phases in the bismuth titanate family have generated great interest in recent years, mainly due to their high temperature stability and high dielectric constants^1^. These make them particularly well suited for a large number of applications such as capacitors, sensors^2^, semiconductors^3^ and piezoelectrics^1,4–6^ as well as lead free high temperature piezoelectrics highly influenced by additives^7–9^.

At least five different crystalline phases are known^10,11^ for the Bi_2_O_3_-TiO_2_ system, but the most frequently studied is the “Aurivillius phase” of the composition Bi_4_Ti_3_O_12_, mainly due to its polarization capabilities. This phase is among the perovskites of the general composition (Bi_2_O_2_)(A_n−1_B_n_O_3n+1_)^12^ and highly affected by the presence of other elements where a large cation with the coordination number 12 (for example Na^+^, K^+^, Ca^2+^, Sr^2+^, Ba^2+^, Pb^2+^, Bi^2+^, Ln^3+^) is in position A, while a small cation with the coordination number 6 (for example Fe^3+^, Cr^3+^, Ti^+^, Nb^5+^, Ta^5+^, W^6+^) is in position B. The electric properties of the pure phase are caused by alternating layers of (Bi_2_O_2_)^2+^ and (Bi_2_Ti_3_O_10_)^3–13^. They are also affected by the oxygen vacancies of the Bi-O connections of the Bi_6p_, Bi_6s_ and O_2p_ localized levels^14^. Some authors have discussed the excellent dielectric properties of another typical bismuth-titanate phase of the composition Bi_2_Ti_2_O_7_ with a cubic pyrochlore structure and applications in high frequency microelectronics^15,16^.

Preparation methods such as sol-gel techniques^17,18^ or thin layer techniques^19,20^ using additives such as Zn and Nb were described. Other authors^21^ concluded that increasing the Ti concentrations in the ceramics via a classical solid reaction resulted in an increase of the dielectric constant at high frequencies. The structure of cubic Bi_2_Ti_2_O_7_ has been described as a classical cubic structure with separate Bi displacement and static O’ domain wall displacement^22^. This structure is similar to that of Zn-containing pyrochlores. Their preparation via mixed oxide routes is difficult because of the required long annealing time and the decomposition of this phase at high temperatures^22^. By contrast, the melt synthesis method used in the experiments presented in the current article enables to reduce or completely eliminate the thermal treatment. Thus different zones of crystallisation can be obtained in dependence on the temperature gradient occurring in the melt while cooling. For the future creation of new functional materials, it will be necessary to combine the properties of multilayer, multiphase and bulk materials.

The above-mentioned properties of Bi_4_Ti_3_O_12_ should be combined with those of the pyrochlore phase Bi_2_Ti_2_O_7_, the cubic Bi_2_SiO_5_, and the residual glass. Furthermore, a preparation procedure enabling the modification of physical properties should be found, especially with respect to the dielectric properties. For example, the occurrence of both Bi_2_SiO_5_ and Bi_4_Ti_3_O_12_ has resulted in excellent dielectric properties^15,23^ advantageous for ferroelectric memory materials and laser components.

The microstructure can be controlled via the synthesis method, resulting in a substantial change in the electrical properties of the bismuth titanate containing materials, including the piezoelectric ones. In this article we describe the microstructures and phases resulting from four different routes of synthesis which lead to varying degrees of orientation alignment for Bi_4_Ti_3_O_12_.

## Results and Discussion

**Figure 1:**
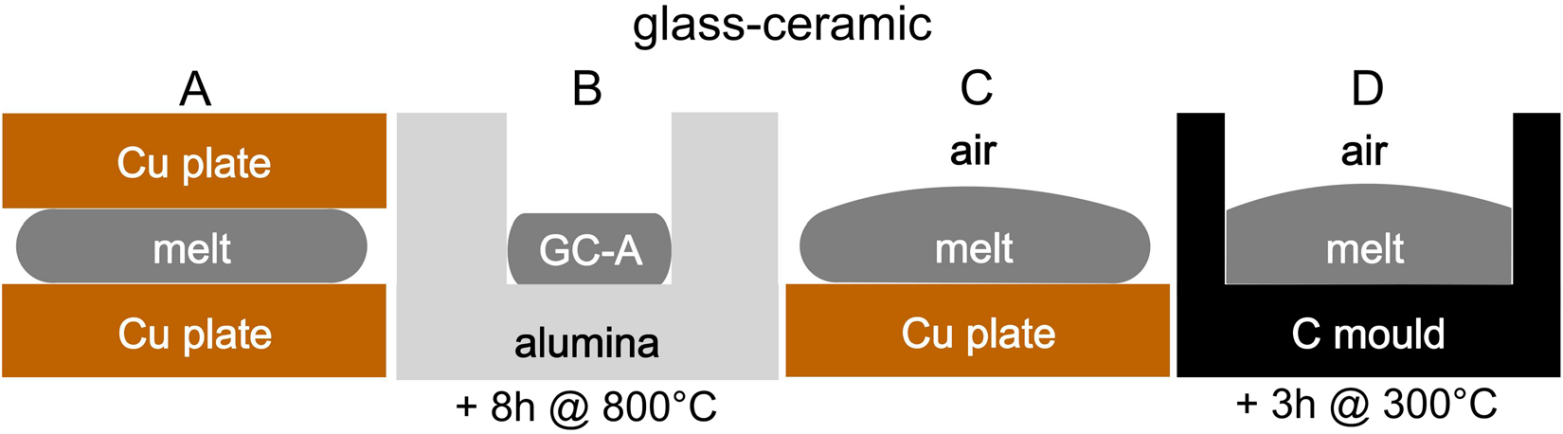
Cooling procedures applied to produce the samples (**A**–**D**).

A glass of the set composition 30 Bi_2_O_3_/50 TiO_2_/10 SiO_2_/10 Nd_2_O_3_ was melted but the composition determined using energy dispersive X-ray spectroscopy (EDXS) after melting was ≈24 Bi_2_O_3_ 40 TiO_2_ 10 SiO_2_ 10 Nd_2_O_3_ 16 Al_2_O_3_ in mol%. Hence this melt must be considered very aggressive towards the Al_2_O_3_ crucibles. The glass was then cooled according to the procedures outlined in Fig. 1: A: quenched to a thickness of 1 mm between two Cu plates, B: part of sample A subsequently heated to 800 °C in an alumina crucible (1 mm wall thickness) with a rate of 7 K/min where it was held for 8 h, C: pouring the melt on a Cu plate so that the final thickness is again 1 mm and letting it cool to room temperature (RT) and finally D: casting the melt into a graphite mould preheated to 300 °C so that it is filled to a thickness of 8 mm before transferring it to a cooling furnace for 3 h at 300 °C before cooling to RT.

**Figure 2:**
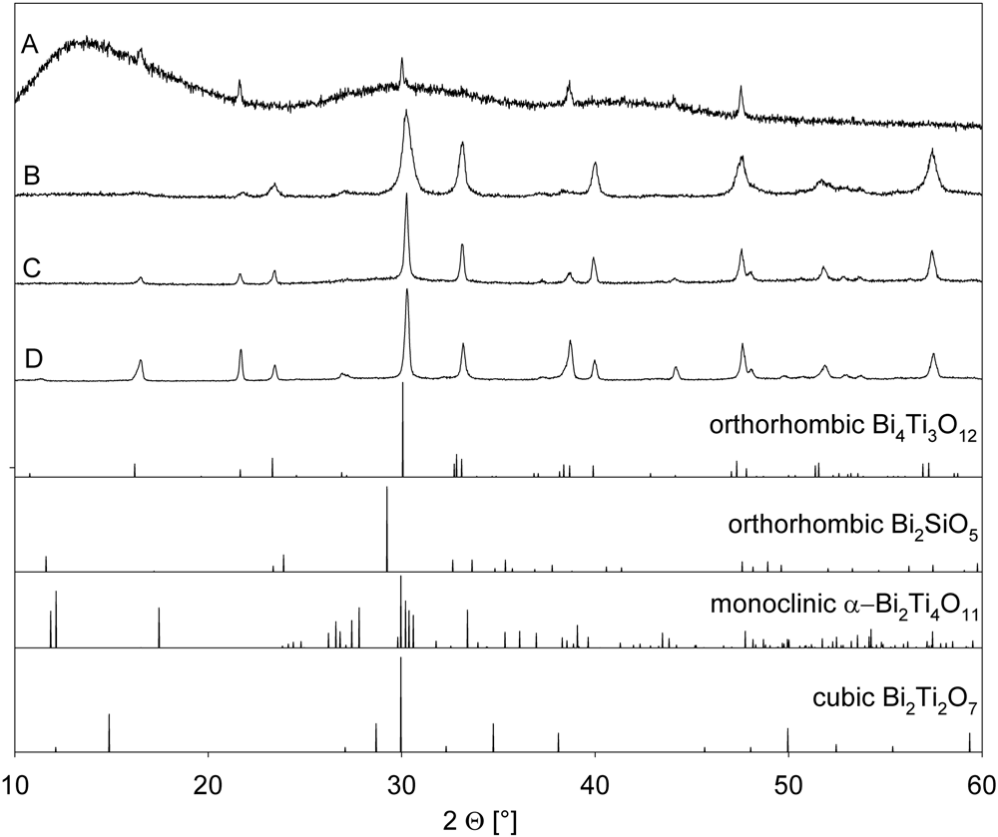
XRD-patterns obtained from powders prepared from the respective glass-ceramics (**A**–**D)**. The theoretical patterns of selected phases are presented below for comparison.

All obtained samples were opaque but a slight translucency was discernible with the naked eye from the middle of a thin cross-section of glass-ceramic A. The samples were first analysed by XRD because previous combined experiments using XRD and EBSD in this system showed that phase analysis is not trivial here^24^. The XRD-patterns A-D in Fig. 2 were acquired from powders prepared from the respective glass-ceramic samples. All peaks are attributable to orthorhombic Bi_4_Ti_3_O_12_ (JCPDS 35-0795). The 100% peak at 2Θ~30°, attributed to the (171) plane, is slightly shifted towards increasingly larger values with the increasing time the samples were held at high temperatures (i.e. from A to D). This indicates that the lengths of the a- and/or c-axes are increasingly reduced while the length of the b-axes remains constant as this shift is not observed for the other peaks. The pattern obtained from the powder of glass-ceramic A additionally contains two broad humps indicating a large amount of amorphous material. Although the other phases featured in Fig. 2 were previously detected in related glass-ceramics^24^, they are clearly not detected by XRD in significant amounts in the samples prepared here.

**Figure 3:**
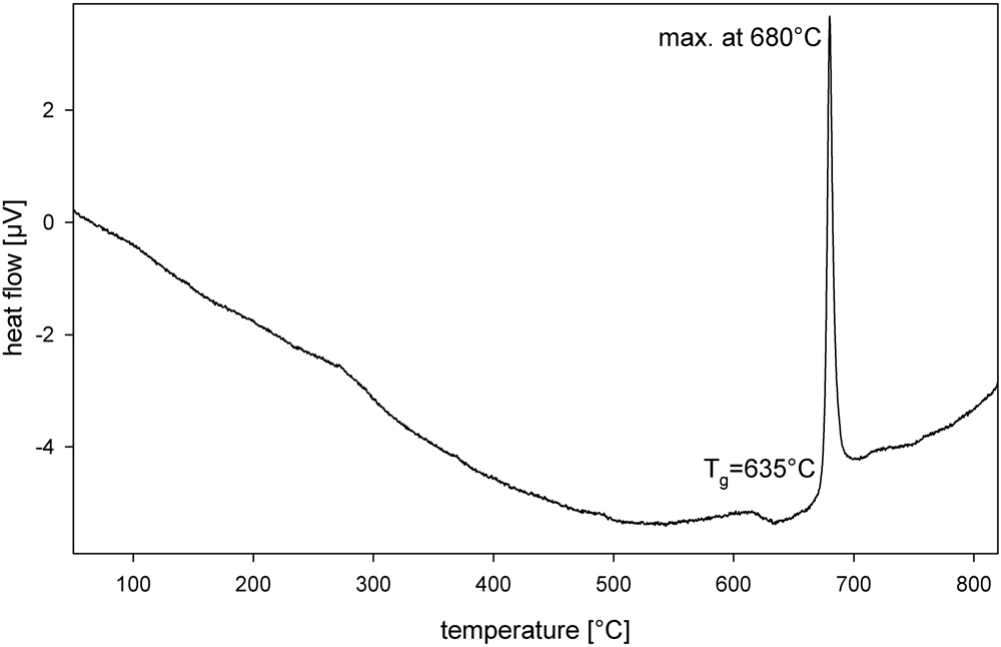
DSC-profile of powdered glass-ceramic A.

As the XRD-pattern of glass-ceramic A indicated a large amount of amorphous material, the powder of this glass-ceramic was analysed by DSC and the resulting profile is shown in Fig. 3: it shows a glass transition temperature T_g_ of 635 °C and a narrow exothermic peak at 680 °C. Hence thermal treatment at 800 °C (sample B) should enable further crystallisation while thermal treatment at 300 °C (sample D) should only slow down the cooling process.

### Microstructure of glass-ceramic A

**Figure 4:**
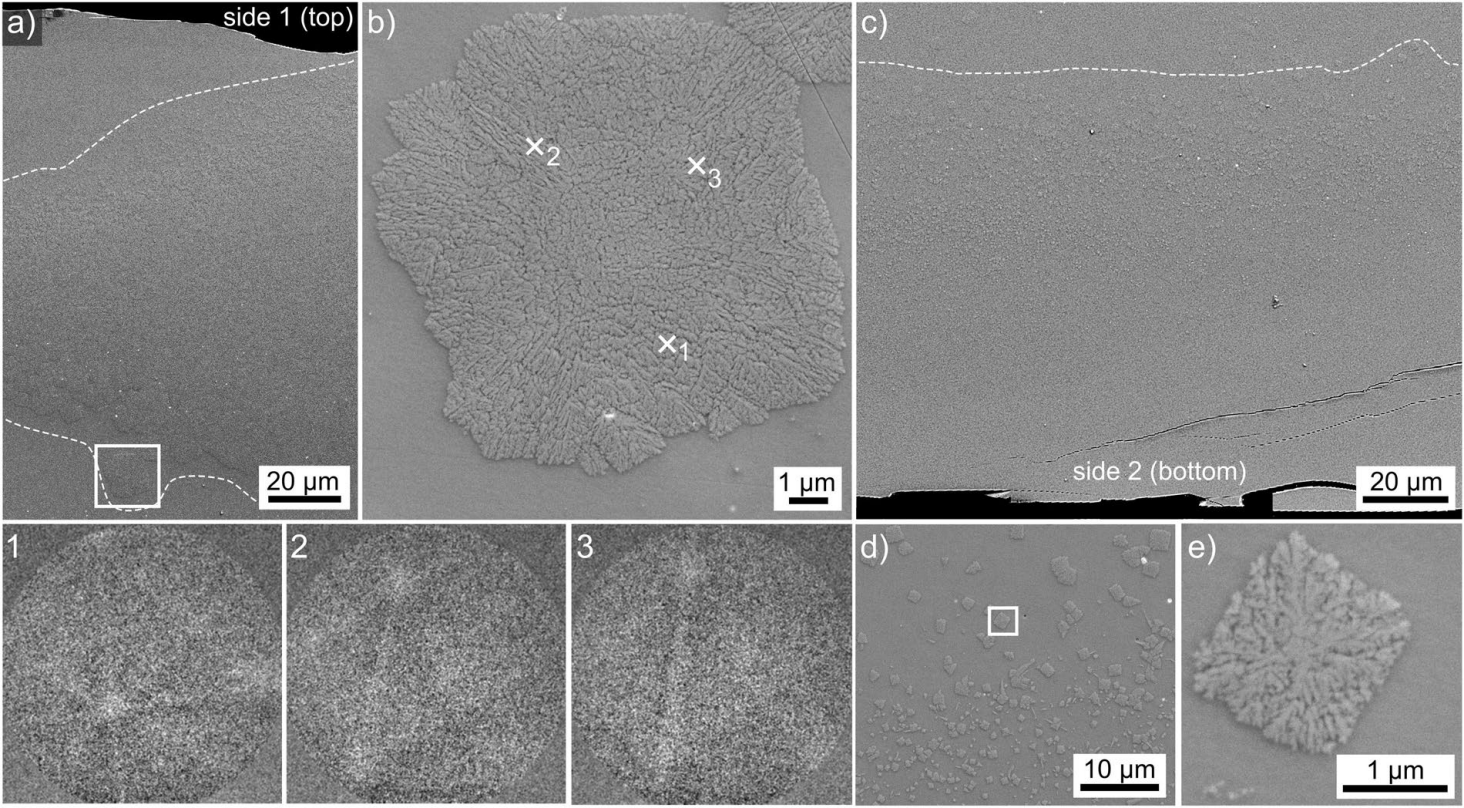
SEM-micrographs illustrating the microstructure in glass-ceramic: (**a**) near the upper Cu/melt interface, the framed area is presented in greater detail (**b**) where the EBSD-patterns 1–3 were acquired at the locations 1–3. (**c**) Overview of the microstructure near the opposite Cu/melt interface, (**d**) in greater detail. The crystal framed here is presented in further detail in (**e**).

The microstructure in glass-ceramic A is illustrated in Fig. 4: an inhomogeneous area of crystallisation, outlined by the dotted lines, occurs near the top of the sample featured in Fig. 4a). The area framed here is presented in greater detail in Fig. 4b to illustrate the fine growth structures observed in this part of the sample. The EBSD-patterns 1–3 were obtained at the locations 1–3 and illustrate the low EBSD-pattern quality obtained from the crystals throughout this glass-ceramic. Although these patterns cannot be indexed due to their low quality, making orientation- or phase analysis by EBSD impossible, they prove the crystallinity of these growth structures and that the growth structure in Fig. 4b contains multiple orientations as pattern 1 clearly differs from the patterns 2 and 3. The SEM micrographs confirm the comparatively low crystallinity indicated by the XRD pattern A in Fig. 2 and are in agreement with the weak translucency observed with the naked eye.

The opposite side of the sample is featured in Fig. 4c where small crystallites are observed. The crystallite size increases with an increasing distance from the Cu/melt interface so that the largest crystals occur at the boundary to the uncrystallised glass in the bulk as shown in Fig. 4d. The area framed here is again presented in greater detail in Fig. 4e in order to show the finely branched microstructure of these growth structures.

Considering the XRD-pattern A in Fig. 2, it is acceptable to conclude that these crystallites are composed of orthorhombic Bi_4_Ti_3_O_12_. EBSD-patterns of similarly low pattern quality have been obtained from Al_2_O_3_-growth structures^25^ where the composition could be verified by TEM^26^. This low pattern quality is probably caused by the finely branched crystals often denoted as the “seaweed morphology” which occurs if dendritic growth is hindered by too low temperatures^27^. The resulting crystal lattice is inhomogeneous, contains tiny pockets of residual glass and hence acquiring high quality EBSD-patterns is improbable.

EDXS-measurements were performed on the glass and the crystallised area shown in Fig. 4b and the obtained compositions are stated in Table 1. All measurements show the same composition within the margin of error, indicating that the growth structures are so fine that the sum of the crystals and the residual glass inside the information volume of EDXS is equal to the composition of the uncrystallised glass.

**Table 1:** Results of EDXS-measurements performed on glass-ceramic A.

mol %	Bi_2_O_3_	TiO_2_	SiO_2_	Nd_2_O_3_	Al_2_O_3_
glass spot	25	40	10	10	15
glass area	24	40	10	10	16
crystal spot	24	40	10	10	16

(margin of error: ±1%).

### Microstructure of glass-ceramic B

**Figure 5:**
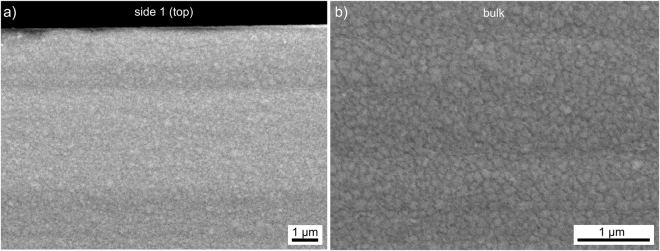
SEM-micrographs featuring the microstructure (**a**) near side 1 and (**b**) in the bulk of glass-ceramic sample B.

Part of the sample featured in Fig. 4 was thermally treated in an Al_2_O_3_ crucible at 800 °C for 8 h in order to produce glass-ceramic B. The microstructure representative for side 1 (the top) is presented in Fig. 5a which shows tiny crystallites. This microstructure is also observed throughout the bulk of the sample as illustrated by Fig. 5b which shows this microstructure in greater detail: the individual structures are ca. 100 nm in diameter. Although indexable EBSD-patterns could not be obtained from this microstructure, probably due to their small size, their homogeneous morphology indicates that these structures were formed by bulk nucleation of the uncrystallised glass with a high nucleation rate in contrast to the growth structures featured e.g. in Fig. 4b. As this microstructure occupies most of the volume in this sample and the corresponding XRD-pattern B in Fig. 2 only shows orthorhombic Bi_4_Ti_3_O_12_, it is plausible to conclude that these structures are composed of this phase.

**Figure 6:**
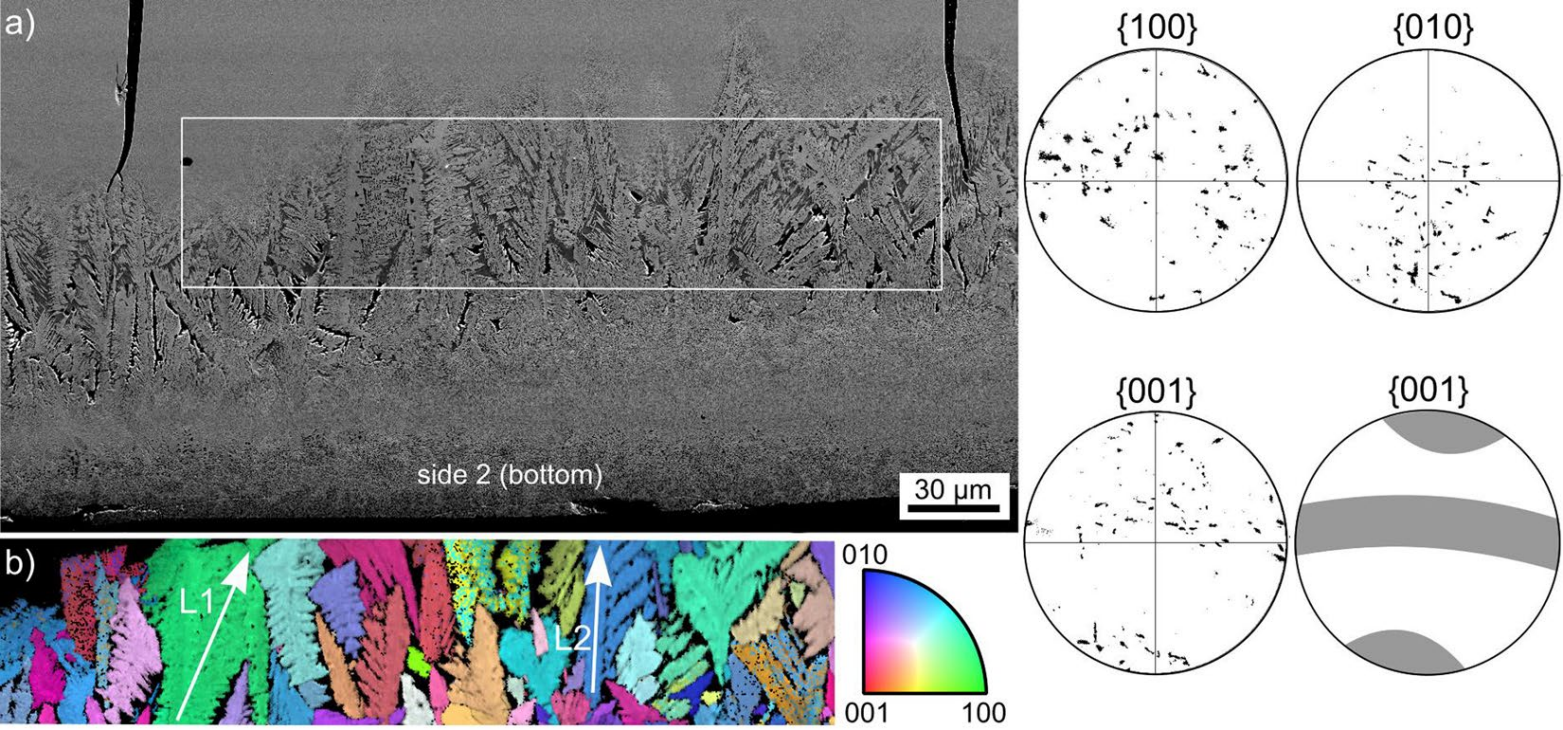
(**a**) SEM-micrograph featuring the microstructure near the bottom side of glass-ceramic B. (**b**) The IPF + IQ-map of an EBSD-scan performed in the framed area. Pole figures illustrating the orientation of Bi_4_Ti_3_O_12_ in the scanned area are presented below.

The microstructure near the opposite side 2 (bottom) of glass-ceramic B is illustrated in Fig. 6a. After ca. 100 µm of fine grained growth, a similarly thick layer of coarse dendrites is observed. The framed area was scanned by EBSD using a step size of 500 nm and the combined inverse pole figure (IPF) and image quality (IQ)-map of the scan is presented in Fig. 6b. As expected of dendritic growth, these dendrites show a very homogeneous orientation, e.g. ~3.0° over 60 µm along the arrow L1 and ~2.5° over 50 µm along arrow L2. Homogeneously oriented Bi_4_Ti_3_O_12_ platelets were also described after molten salt synthesis where dendritic growth did not occur^28^. Pole figures (PFs) illustrating the orientations of the main crystallographic axes of Bi_4_Ti_3_O_12_ in the scanned area are presented below. Considering the large size of the individual orientation domains and hence the few number of “grains”, a texture evaluation is problematic. However, a slight orientation preference may be deducted from the {001}-PF as visualized by the illustrated {001}-PF to the right. If this orientation preference is real, the long a-axis (32.83 Å) would preferably occur parallel or perpendicular to the growth direction.

**Figure 7:**
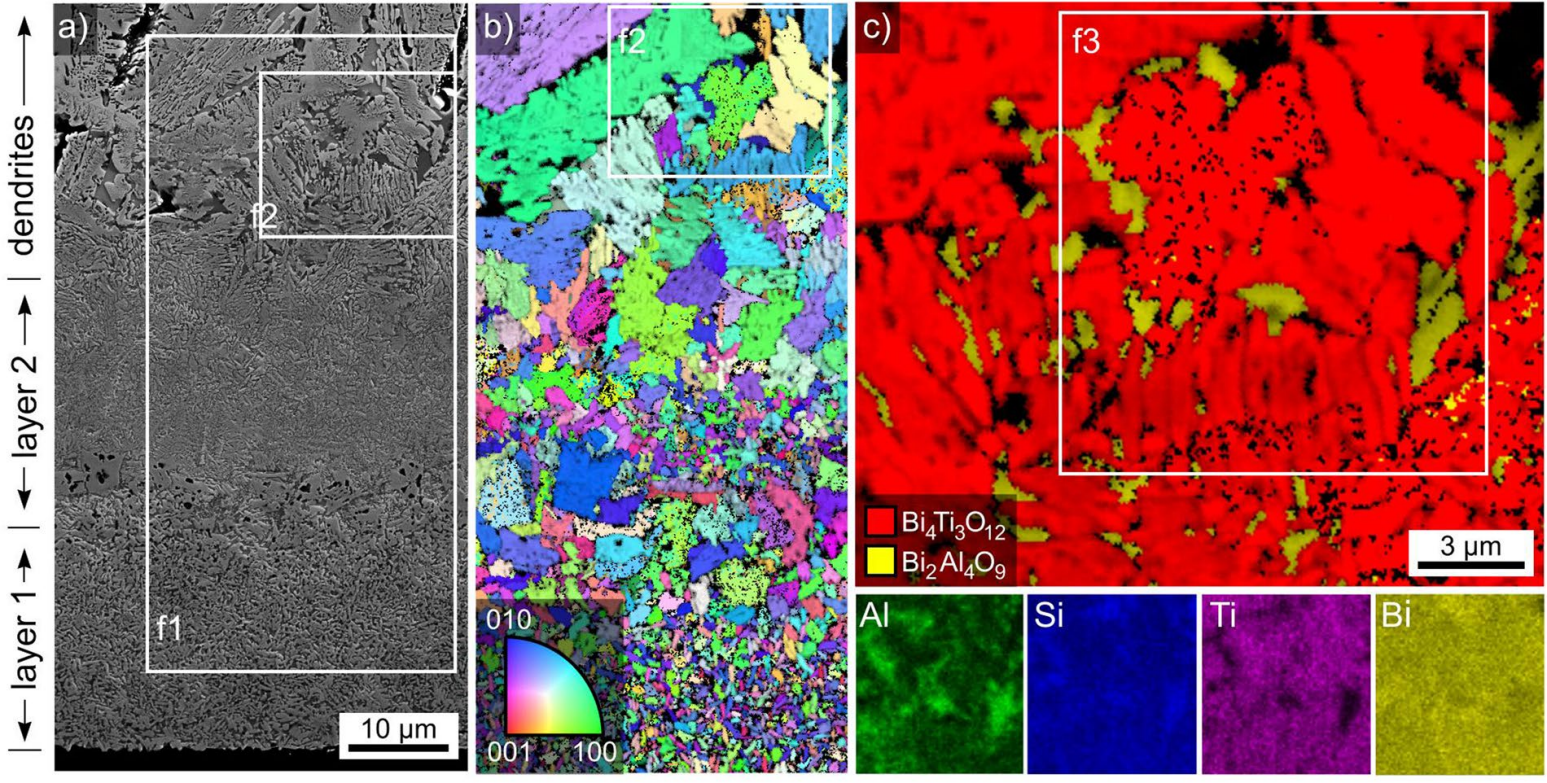
(**a**) SEM-micrograph showing the fine crystallisation at the bottom edge of glass-ceramic B. (**b**) IPF + IQ-map of an EBSD-scan performed in frame f1. (**c**) Phase map of the EBSD-scan in frame f2 and element maps of Al, Si, Ti and Si in the area of frame f3.

The fine grained microstructure below the layer of dendrites is presented in greater detail in Fig. 7a where layer 1 shows smaller crystals than layer 2. This impression is confirmed by the IPF + IQ-map in Fig. 7b of an EBSD-scan performed on the area inside frame f1. Clear orientation preferences could not be detected for Bi_4_Ti_3_O_12_ in either of the layers. The phase map of the EBSD-scan inside the frame f2 is presented in Fig. 7c in order to illustrate that some data points acquired in between the Bi_4_Ti_3_O_12_ crystals are reliably indexed as orthorhombic Bi_2_Al_4_O_9_ using a material file based on ICSD-file no. 20069. Most of the Bi_2_Al_4_O_9_-domains in Fig. 7c share a common orientation with a tolerance of 5°, indicating that they originate from a common nucleus and grew along the existing channels of residual glass, similar to the behaviour of cordierite after the crystallisation of multiple yttrium silicates^29^ or fresnoite after the crystallisation of BaTiO_3_^30^ in glasses. An epitaxial relationship similar to that observed between Bi_4_Ti_3_O_12_ and Bi_2_SiO_5_ in related glass-ceramics^24^ could not be discerned.

The element maps of Al, Si, Ti and Bi obtained via EDXS are presented below. The area where Bi_2_Al_4_O_9_ is observed in Fig. 7c is enriched in Al and Si but depleted of Ti while the Bi-map does not show a strong contrast. Similar observations are made for areas where reliably indexable EBSD-patterns could not be acquired and which are most probably occupied by residual glass.

Chemical compositions determined from EDXS spot measurements at various locations are presented in Table 2. The chemical composition determined at a location where the EBSD-patterns were indexed as Bi_2_Al_4_O_9_ in Fig. 7c shows a high amount of Al instead of Si, confirming that this phase is a Bi-Al-O compound. The composition of the residual glass determined at two independent locations is identical and shows that it mainly contains Bi, Si and Al. The composition measured for the microstructure in Fig. 5b basically matches the composition determined for the uncrystallised glass in Table 1 but in at%.

**Table 2:** Results of EDXS-measurements performed on glass-ceramic B.

at %	Bi	Ti	Si	Nd	Al	O
indexed as Bi_2_Al_4_O_9_	9	2	7	1	26	55
ideal Bi_2_Al_4_O_9_	13	0	0	0	27	60
residual glass1	16	3	13	2	9	57
residual glass2	16	3	13	2	9	57
crystals in Fig. 5b	14	11	3	4	11	57

(margin of error: ±1%).

The microstructure described for glass-ceramic B may be explained by considering that this sample was produced by annealing part of glass-ceramic A at 800 °C, i.e. far above the T_g_ of the glass, inside an alumina crucible placed inside a furnace. This setup leads to a strong thermal gradient in the sample because the bottom (side 2) is in contact with alumina while the top (side 1) is in contact with air. At the same time, the crucible initially shields the sample from thermal radiation. Hence the pre-crystallised area outlined in Fig. 4c was probably heated before the bulk of the uncrystallised glass reached the nucleation temperature. Hence the pre-existing crystals continued to grow and those adjacent to the bulk of uncrystallised glass switch to the dendritic growth mechanism as the growth velocity increases with an increasing temperature. This growth is able to continue until the bulk of the glass reaches the nucleation temperature and the bulk crystallisation illustrated in Fig. 5 blocks further growth.

Due to the longer annealing time and the higher degree of crystallinity, larger pockets of residual glass of a modified composition form in between the Bi_4_Ti_3_O_12_ crystals, allowing the formation of Bi_2_Al_4_O_9_ via secondary crystallisation. The high amount of Al_2_O_3_ from the crucible is accumulated in this residual glass and probably stabilized it against crystallisation as Al is a good network former and should lead to a notable increase in viscosity.

### Microstructure of glass-ceramic C

**Figure 8:**
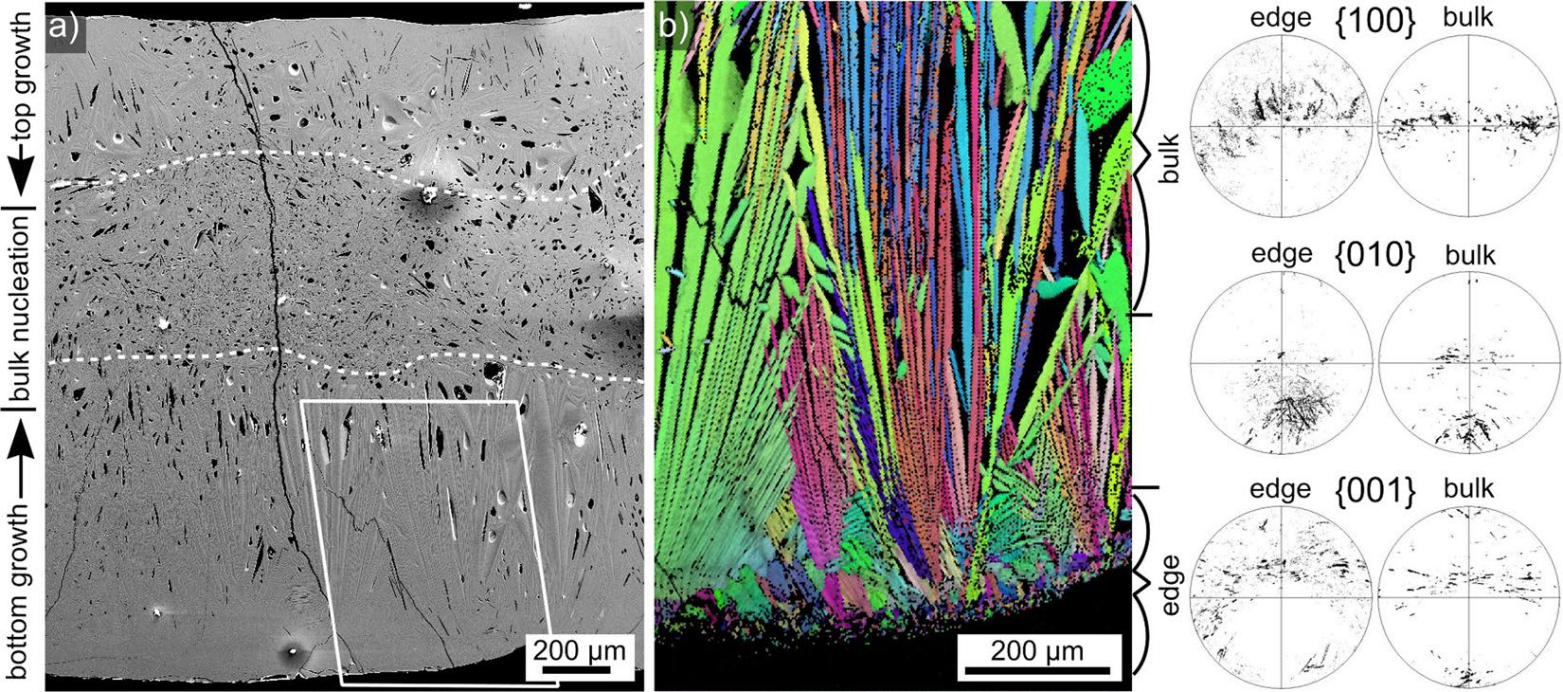
(**a**) SEM-micrograph of the entire cross section of glass-ceramic C. An EBSD-scan was performed in the framed area. (**b**) IPF + IQ-map of the EBSD-scan as well as 100, 010 and 001 PFs illustrating the orientation of the respective crystallographic axes of Bi_4_Ti_3_O_12_ in the highlighted sections near the edge and in the bulk.

Glass-ceramic C was produced by simply pouring the melt onto a Cu plate, i.e. quickly cooling the bottom of the sample while the top cooled much slower via the contact to air. The resulting microstructure is illustrated in Fig. 8a which shows a complete cross section of sample C. While a relatively compact glass-ceramic is observed at the top and bottom interfaces, large pores are increasingly observed in the bulk. Growth structures originating from the interfaces grow into the bulk to form a ca. 900 µm thick layer at the bottom while the layer at the top is only half as thick.

An EBSD-scan was performed in the area framing the aligned growth structures in the bottom layer and showed them to be composed of orthorhombic Bi_4_Ti_3_O_12_ in agreement with the corresponding XRD-pattern C presented in Fig. 2. Other crystalline phases were not detected in this sample. The IPF + IQ-map of this scan is presented in Fig. 8b and PFs outlining the orientations of the main crystallographic axes near the edge and further in the bulk are presented: the {100} PFs show a clear preference of orientations with their long a-axis perpendicular to the main growth direction as well as a growth selection enhancing this preference during growth into the bulk in the scanned area. Textures were not detected in EBSD-scans performed in the bulk, supporting the assumption that the crystals here formed without a reference system comparable to the top and bottom interfaces.

**Figure 9:**
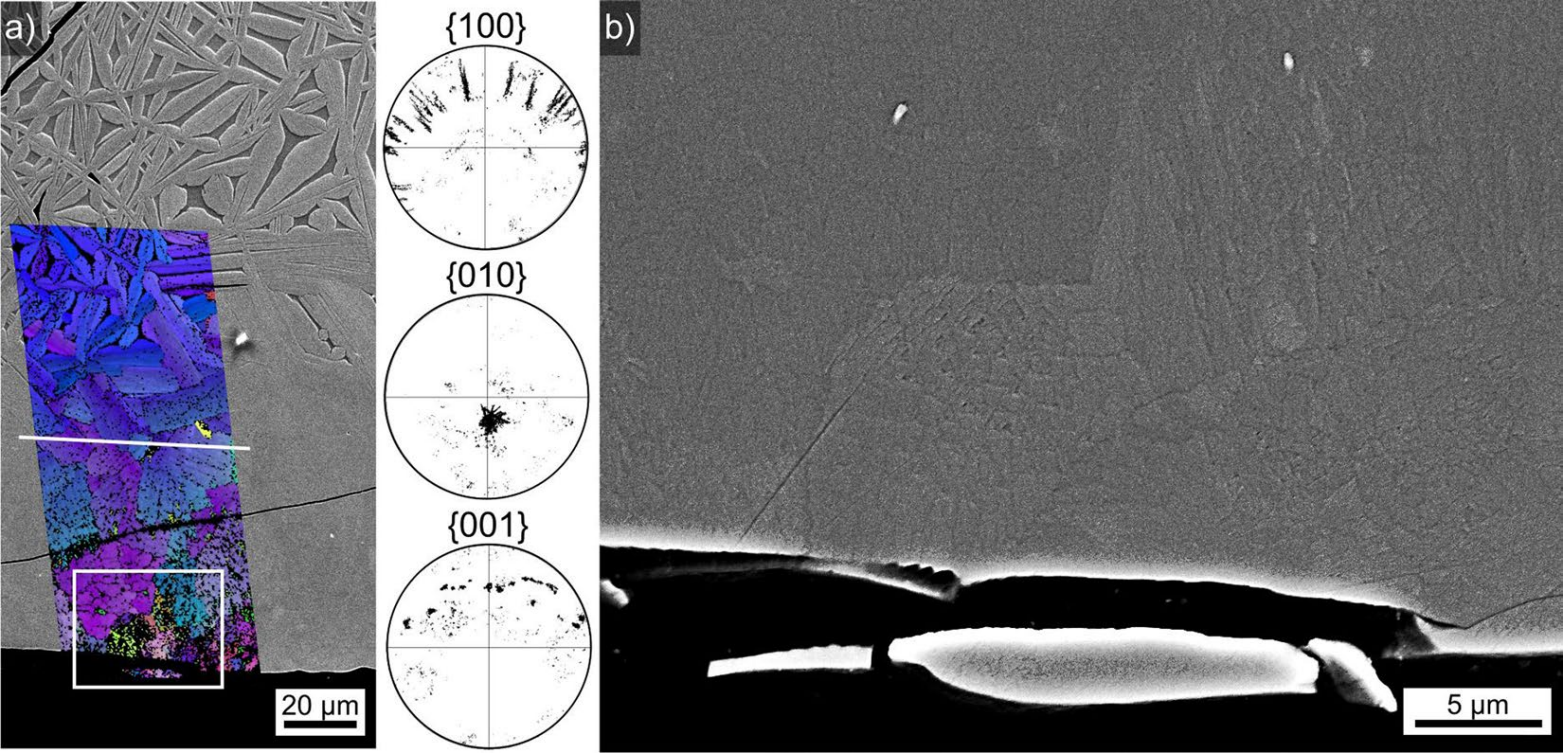
(**a**) SEM-micrograph of the immediate bottom edge of glass-ceramic C superimposed by the IPF + IQ-map of an EBSD-scan performed on the area. The 100, 010, and 001 PFs of the data points below the white line are also presented. (**b**) An SEM-micrograph of the framed area.

The microstructure at the immediate interface to the Cu-plate (bottom) is presented in Fig. 9. The first ca. 60 µm of growth, probably at a temperature just above T_g_, lead to such a fine microstructure that barely any contrast occurs in the SEM-micrographs. The presented PFs indicated that the b-axis of the crystals in the scanned area show very similar orientations while the a- and b-axes do not. Additionally, systematic orientation shifts of the a-axes occur while the positions of the c-axes remain constant.

Chemical compositions determined from EDXS spot measurements at various locations are presented in Table 3. Si and Al are similarly enriched in the residual glass found in between the large crystals.

**Table 3:** Results of EDXS-measurements performed on glass-ceramic C.

at %	Bi	Nd	Ti	O	Si	Al
Large crystal bulk	13	10	17	59	0	1
Large crystal top	17	5	15	58	3	2
Small crystal bottom	17	6	14	57	3	3
residual glass	18	1	5	57	10	9

(margin of error: ±1%).

The presented results show that a strong thermal gradient results in large plates of Bi_4_Ti_3_O_12_ growing from the cooled interface into the hot bulk until the latter becomes cold enough to allow bulk nucleation. The observation of large areas of such aligned crystals at the Cu/glass interface illustrates the potential to achieve the growth of macroscopically thick layers of oriented orthorhombic Bi_4_Ti_3_O_12_ in this glass.

### Microstructure of glass-ceramic D

**Figure 10:**
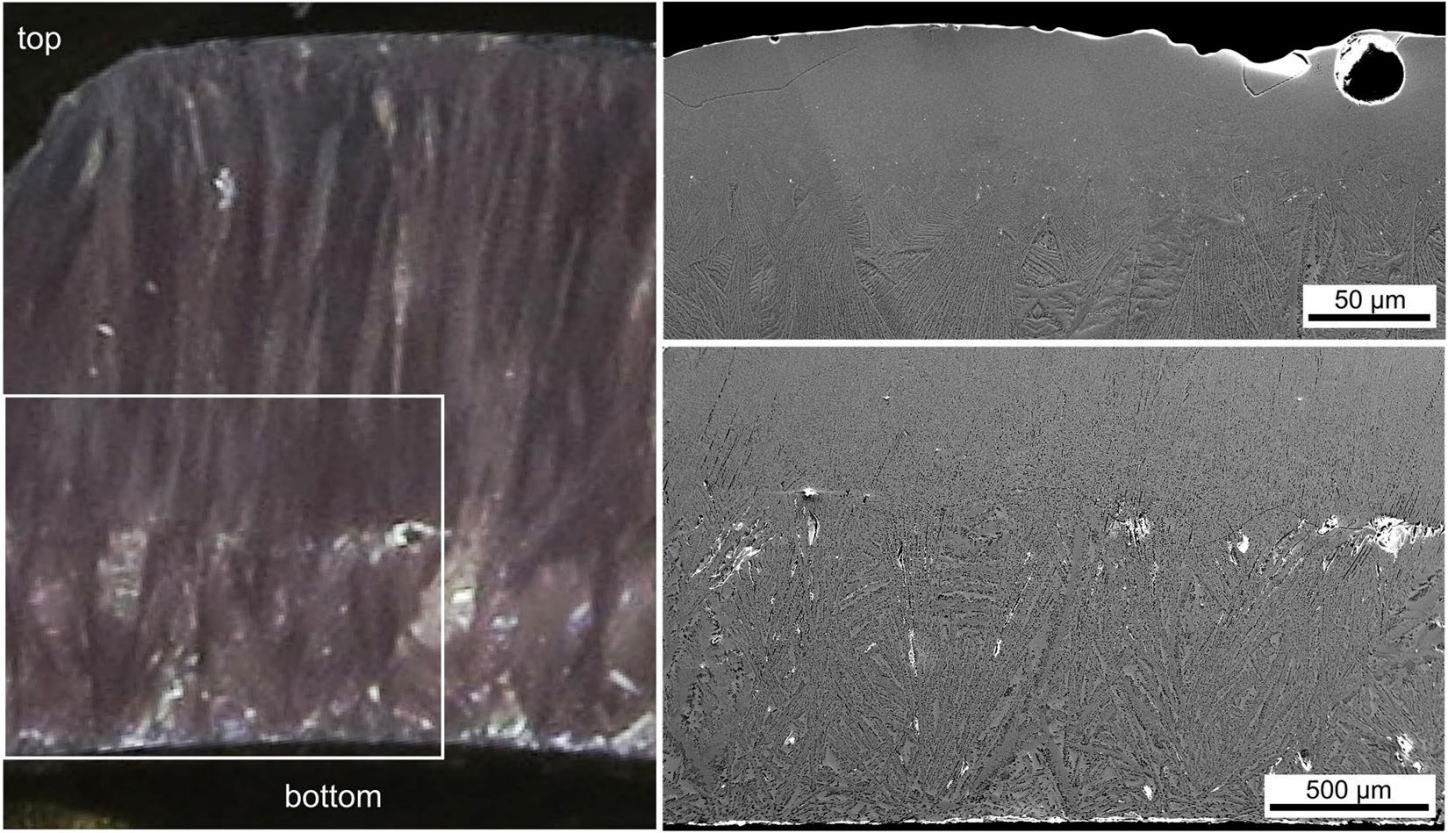
Photograph of the cross section of sample D. SEM micrographs present the top edge and the framed area in greater detail.

Glass-ceramic D was produced by pouring the melt into a preheated graphite mould, hence this sample was cooled more slowly than the described samples above with an air/glass interface at the top and glass/graphite interfaces at the bottom and the sides. The photograph of a cross section of this sample presented in Fig. 10 implies two layers of growth: a thicker layer of growth from the top and a thinner layer of growth from the bottom.

**Figure 11:**
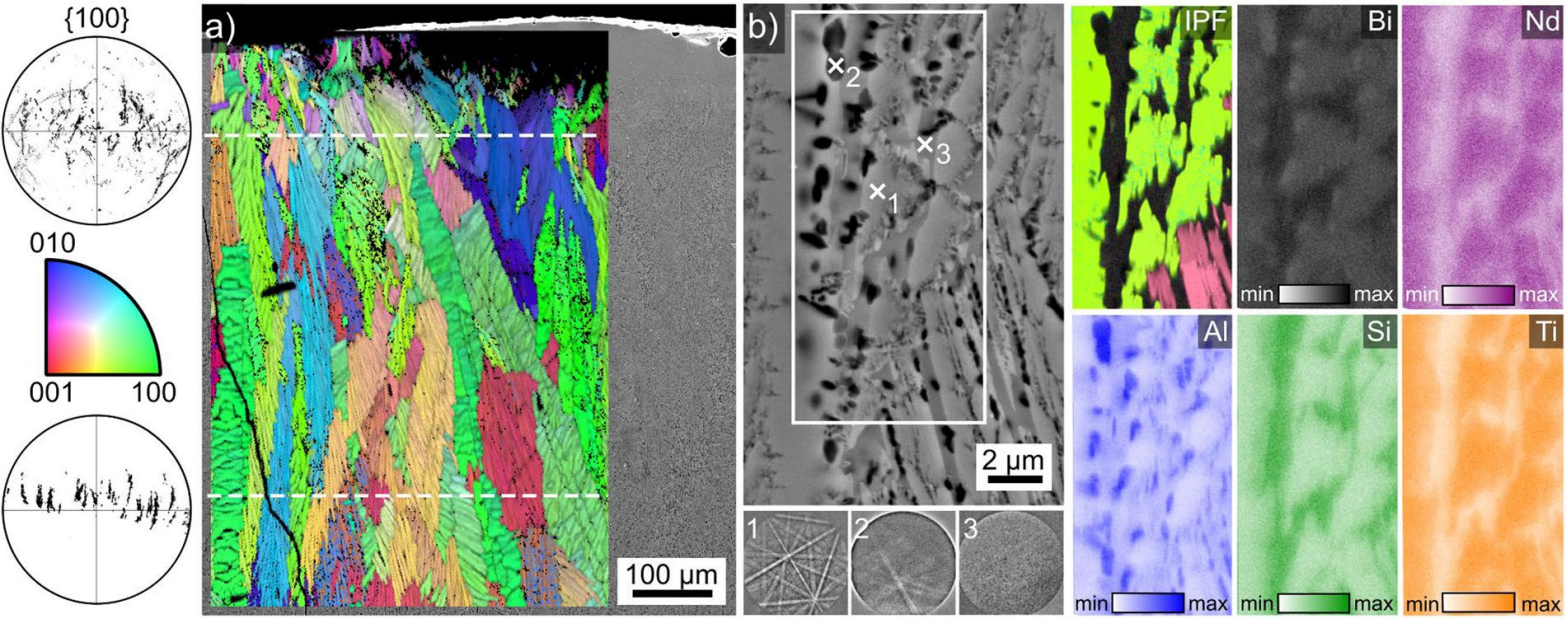
(**a**) SEM-micrograph showing the air/glass-interface of glass-ceramic D superimposed by the IPF + IQ-map of an EBSD-scan performed on the area. 001 PFs of the scan areas above the upper and below the lower dashed lines are presented. (**b**) SEM-micrograph of the detailed microstructure along with the IPF + IQ-map and the element maps of Bi, Nd, Al, Si and Ti obtained within the framed area. The EBSD-patterns 1–3 were acquired at the locations 1–3.

An SEM-micrograph of the air/glass-interface superimposed by the IPF + IQ-map of a performed EBSD-scan is presented in Fig. 11a, all reliably indexed EBSD-patterns were attributed to orthorhombic Bi_4_Ti_3_O_12_. While EBSD-data could be acquired near the immediate surface in the left part of the scan, there is a layer just below the surface where data could not be acquired in the right part of the scan. The size of the homogeneously oriented domains increases with a growing distance from the surface, implying a kinetic selection. This impression is supported by the presented 100 PFs representing the orientation data above the top dashed line and below the bottom dashed line: only orientations with the long a-axis oriented perpendicular to the primary direction of growth remain in the bulk.

The detailed microstructure amongst the large orientation domains in the bulk is illustrated in Fig. 11b. The SEM-micrograph contains features of a bright grey, black and dark grey material contrast. The EBSD-patterns 1–3 acquired from these areas show that the bright grey areas provide high quality EBSD-patterns which are indexable as orthorhombic Bi_4_Ti_3_O_12_. The black areas provide low quality patterns while the dark grey areas provide no diffraction patterns at all, i.e. they are probably amorphous as the sample preparation is obviously acceptable for EBSD analysis as proven by the patterns 1 and 2. The IPF + IQ-map shows that the Bi_4_Ti_3_O_12_ crystals in the framed area belong to two orientation domains. The black areas in the SEM-micrograph are enriched in Al, see element map of Al, and failed to provide acceptable EBSD-patterns during the scan because they have a much lower backscatter efficiency and require a longer pattern acquisition time than was provided during the scan. The dark grey amorphous phase, i.e. the residual glass, is enriched in Bi and Si but depleted of Nd and Ti as illustrated by the respective element maps.

EDXS results obtained from this glass-ceramic are presented in Table 4. The black structure marked by x2 in Fig. 11b provided a composition containing mainly Al and O, but also Bi and Ti. Due to its small size, the information volume providing this result likely contains at least one further phase. The composition measured from a larger structure of the same SEM-contrast shows the almost ideal composition of Al_2_O_3_. The composition of the residual glass measured at location x3 in Fig. 11b is also stated. Comparing the latter with the results obtained from an area scan, i.e. an approximation of the melt before it crystallised, it confirms the impression gained from the element maps in Fig. 11b: the residual glass is enriched in Bi_2_O_3_ and SiO_2_ but depleted of the remaining elements.

**Table 4:** Results of EDXS-measurements performed on glass-ceramic D: spot measurements as well as an area scan.

at %	Bi	Nd	Ti	Si	Al	O
black (X2)	9	1	4	3	27	56
black (larger)	1	2	1	1	41	54
ideal Al_2_O_3_	0	0	0	0	40	60
mol %	Bi_2_O_3_	Nd_2_O_3_	TiO_2_	SiO_2_	Al_2_O_3_	
res. glass (X3)	31	6	24	23	16	
area scan	24	9	38	9	20	

(margin of error: ±1%).

**Figure 12:**
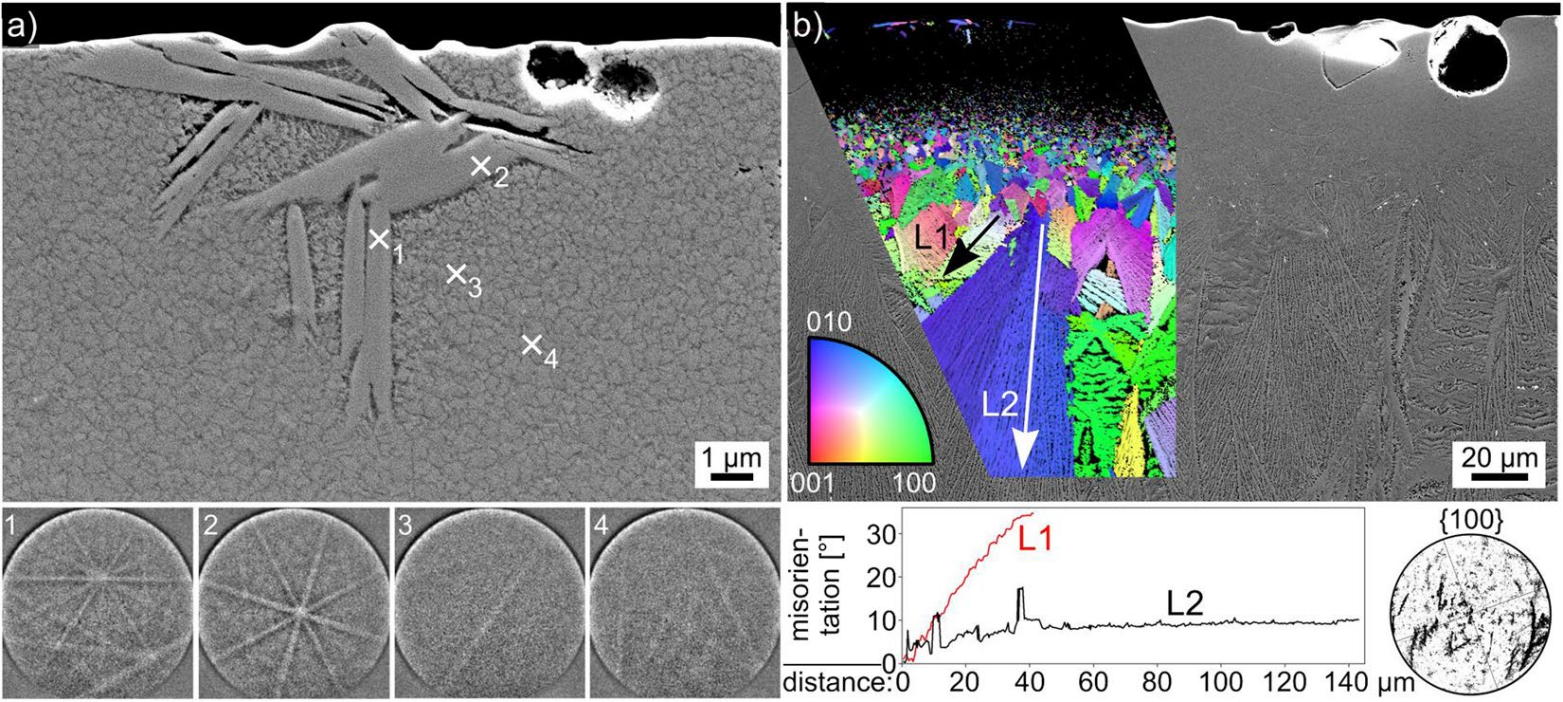
(**a**) SEM-micrograph of the immediate air/glass interface of sample D. The EBSD-patterns 1–4 were acquired at the locations 1–4. (**b**) SEM-micrograph providing an overview of the same interface superimposed by the IPF + IQ-map of an EBSD-scan performed on the area. The misorientations along the lines L1 and L2 are presented below as well as the 100-PF of the scan.

The microstructure in the area where EBSD-patterns could not be acquired adjacent to the surface in Fig. 11a is illustrated in Fig. 12. The immediate interface presented in Fig. 12a shows a few larger crystals embedded in a matrix of sub-µm sized grains. The EBSD-patterns 1–4 recorded at the locations 1–4 illustrate the different pattern qualities obtained from these crystals: the large crystals provide reliably indexable EBSD-patterns while the pattern quality obtained from small crystals is generally too low for indexing. Figure 12b features this interface with a lower magnification to show that the grain size increases with an increasing distance from the surface. The superimposed IPF + IQ-map of an EBSD-scan shows that reliably indexed EBSD-patterns were only obtained from the larger crystals at the immediate interface and then from the increasingly large growth structures in the bulk. The growth structures adjacent to the fine grained layer resemble those also described in Fig. 7b, i.e. individual crystallites with independent orientations.

At some distance from the surface, the crystal growth mechanism becomes dendritic and it must be noted that continuous orientation changes inside individual dendrites occur close to the surface while the large orientation domains observed further in the bulk show a more homogeneous orientation. The orientation change along line L1 shows a misorientation of 20° over a distance of only 15 µm while the misorientation along line L2 is only 6° over a distance of 100 µm. This is important because dendrites generally show a homogeneous orientation but e.g. continuous orientation changes in dendrites were also observed during the non-isothermal crystallisation of Sr-fresnoite^31^. Here the systematic orientation change was interpreted to correspond to a change of the growth mechanism from dendritic to what could be viscous fingering^31^ during cooling. In the sample presented here, the microstructure and the orientation analysis indicate an increasing temperature during crystal growth with an increasing distance from the air/glass interface. A high nucleation rate leads to the fine grained microstructure near the interface and then increasingly larger crystallites including dendrites with orientation changes until basically homogeneously oriented plates grow into the bulk. The large orientation domains grown at the highest temperature rather appear as parallel plates than classic dendrites, but as they share a common orientation, it seems plausible that the more delicate features of classic dendritic growth are prevented by the high temperature of the matrix.

An overall alignment of the crystals in the scanned area is indicated in the 100 PF of the scan presented in Fig. 12b. The “streaks” in the PF result from continuous orientation changes in certain dendrites as outlined above. As all the “streaks” are more or less parallel to each other, it may be concluded that the orientation shift inside the independent dendrites occurs systematically and due to the same diving force. It is probable that they shift towards an optimal growth direction at a certain temperature so as to enhance the growth velocity. As the crystals proceed to grow into the hotter part of the sample, this adaptation is no longer needed and classical, homogeneous dendritic growth is observed due to the higher diffusivities.

**Figure 13:**
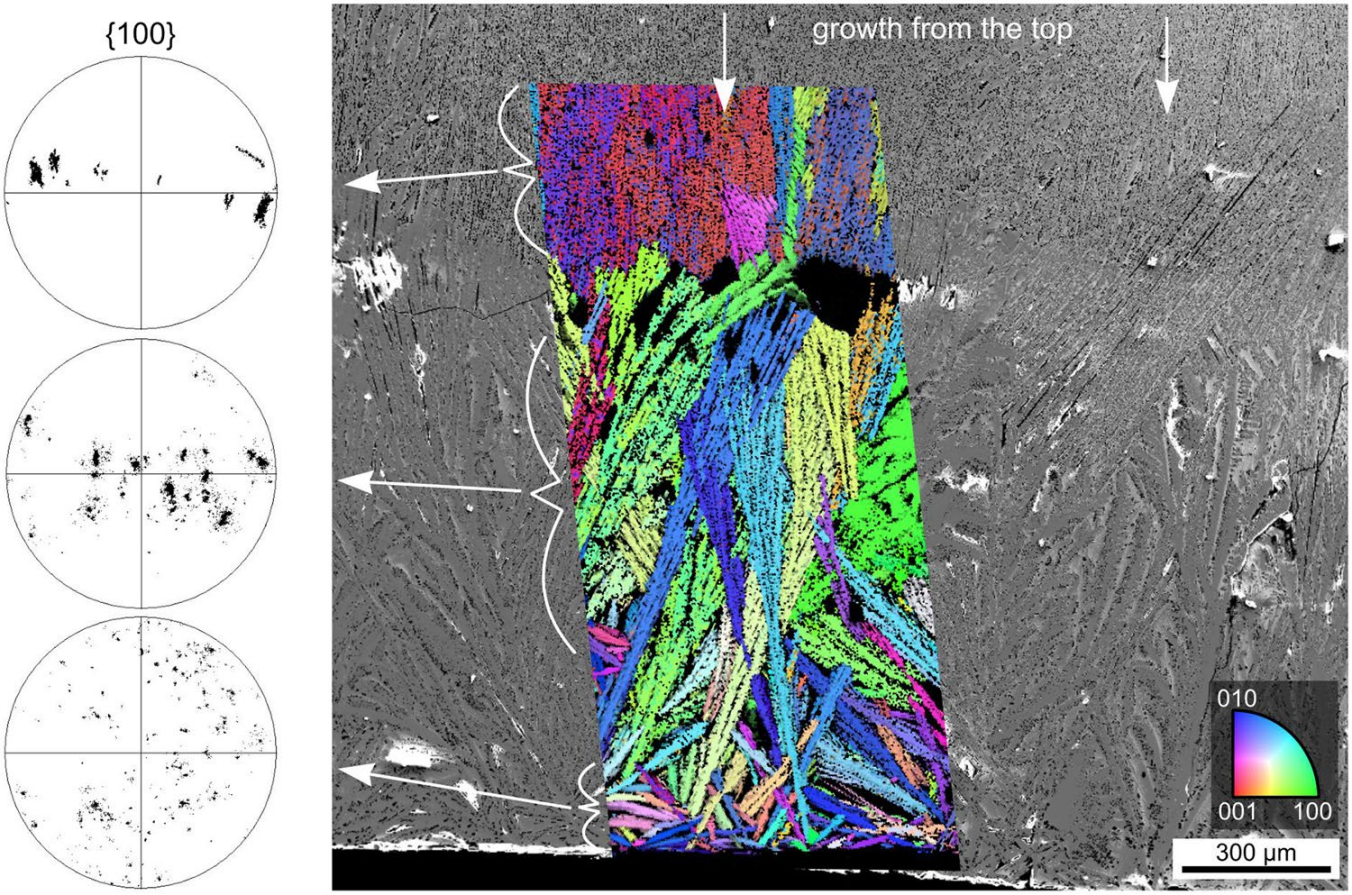
SEM-micrograph featuring the bottom interface of glass-ceramic D as well as the area where the growth fronts from the opposing interfaces of the sample collided. It is superimposed by the IPF + IQ-map of an EBSD-scan performed on the area and the 100 PFs of the respectively highlighted domains of growth are presented.

An SEM-micrograph superimposed by the IFP + IQ-map of an EBSD-scan performed on the glass/graphite interface on the bottom of the sample is shown in Fig. 13. While plates of various orientations are observed at the immediate interface (bottom), they evolve into larger orientation domains during growth into the bulk until they collide with the crystallisation front growing from the top of the sample, i.e. bulk nucleation is not observed. Al_2_O_3_ crystals similar to those featured in Fig. 11b also occur but do not contribute to the EBSD-scan due to their low pattern quality. The presented {100}-PFs of Bi_4_Ti_3_O_12_ show that the same growth selection observed at the top of the sample also occurs at the bottom so that ultimately two (100)-oriented layers of Bi_4_Ti_3_O_12_ collide in the bulk.

### d_33_-measurements

**Figure 14:**
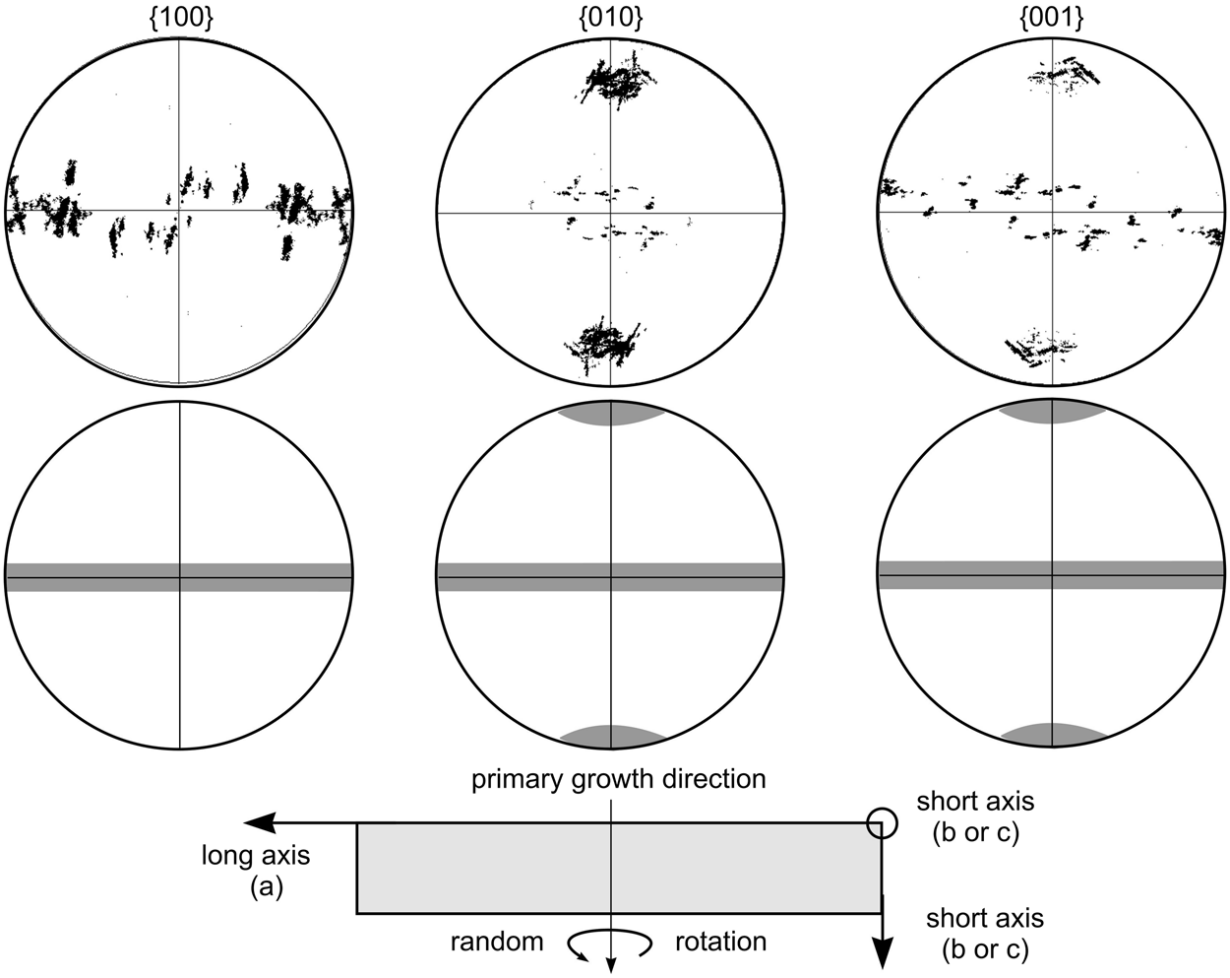
100, 010 and 001 PFs of Bi_4_Ti_3_O_12_ (poles in both hemispheres) in the bottom part of the scan also featured in Fig. 11 (**a**). Idealized textures are presented below as well as the resulting orientation preference of the corresponding unit cell in the glass-ceramic.

Due to the lack of bulk nucleation, the sample preparation for glass-ceramic D is suitable to produce macroscopically thick layers of oriented orthorhombic Bi_4_Ti_3_O_12_. As the unit cells of the Bi_4_Ti_3_O_12_ crystals show variable attributions of the longest lattice parameter to one of the crystallographic axes in the literature, the properties cannot be discussed in terms of crystallographic axes. The texture described for glass-ceramic D shows the short axes either perpendicular or parallel to the surface. Hence, the long crystallographic axis is always parallel to the surface. This is confirmed by the PFs in Fig. 14 and the idealized textures illustrated below them. The preferred orientations of the unit cell are illustrated below: the shorter axes basically show the same textures because if either of them is perpendicular to the surface, the other is parallel to it.

Values for the spontaneous polarization of Bi_4_Ti_3_O_12_ have been reported as 4 µC/cm^2^ along the c_0_-direction and more than 30 µC/cm^2^ along the b_0_-direction, albeit in a crystal described to be monoclinic and containing complex twinning^11^.

A roughly 4 mm thick sample of an oriented layer of Bi_4_Ti_3_O_12_ in glass-ceramic D (i.e. from one half of the produced glass-ceramic body) was produced, polished and contacted by applying the layer of Au via sputtering. This contacted layer of oriented Bi_4_Ti_3_O_12_ was then placed in a d_33_-meter but failed to show any signal of piezoelectricity. As both the orientation alignment as well as the piezoelectric activity of the crystals has been proven, this indicates that the opposing directions of polarization occur statistically, i.e. parallel and anti-parallel orientation have the same probability and hence cancel each other out. Hence these oriented glass-ceramics must be poled in order to achieve a macroscopic piezoelectric effect. Nevertheless, a high degree of orientation of the polar axis should result in higher piezoelectric constants than in statistically oriented samples after both are poled.

It should be noted that polar crystallisation of glass ceramics has already been reported for fresnoites^32^, where poling is hence not required to achieve piezoelectric samples. By contrast, the crystallisation of stuffed β-quartz did not lead to piezoelectric samples^33^. It should be noted that both fresnoite and β-quartz are not ferroelectric and hence cannot be poled.

In summary, all four experimental approaches lead to the crystallisation of the Aurivillius phase Bi_4_Ti_3_O_12,_ but its growth mechanism varies in dependence of the occurring thermal gradients and the resulting crystal growth velocities. Crystallographically oriented layers of Bi_4_Ti_3_O_12_ may be produced, but they must be poled in order to achieve a macroscopic piezoelectric effect.

The prepared melt is highly corrosive towards Al_2_O_3_ crucibles and the secondary phases of Bi_2_Al_4_O_9_ and Al_2_O_3_ were detected in between the Bi_4_Ti_3_O_12_ crystals of the samples B and D respectively.

## Methods

A glass was melted in batches of 100 g using the starting materials Bi_2_O_3_, TiO_2_ (rutile) SiO_2_ (quartz) and Nd_2_O_3_ in alumina crucibles (diameter = 27 mm, height = 30 mm, wall thickness = 2 mm) at 1450 °C for 20 min. The set composition was 30 Bi_2_O_3_/50 TiO_2_/10 SiO_2_/10 Nd_2_O_3_ but the composition determined using energy dispersive X-ray spectroscopy (EDXS) after melting was ≈24 Bi_2_O_3_ 40 TiO_2_ 10 SiO_2_ 10 Nd_2_O_3_ 16 Al_2_O_3_ in mol%. The glass was then cooled according to the procedures outlined in Fig. 1. The Cu plates had a diameter of 100 mm and a thickness of 15 mm. The corundum crucible had external dimensions of 27 mm in diameter and 30 mm in height with a wall thickness of 2 mm. The C-mould (10 mm diameter, 8 mm deep) was drilled from a compact block of C (120 mm diameter, 40 mm thick).

The resulting glass-ceramics were characterized by X-ray diffraction (XRD) using a Phillips APD-15 diffractometer with Cu-Kα radiation and a 0.2° step size in a θ-2θ Bragg-Brentano geometry. The microstructure was analysed by scanning electron microscopy (SEM) using a Jeol JSM 7001 F equipped with an EDAX Trident analyzing system containing a Digiview 3 EBSD-camera. Energy-dispersive X-ray spectroscopy (EDXS) was performed without a standard using an acceleration voltage of 15 kV. EBSD-scans were performed using a voltage of 20 kV and a current of ca. 2.40 nA. The scans were captured and evaluated using the software TSL OIM Data Collection 5.31 and TSL OIM Analysis 6.2. Unreliable data points were removed in all datasets used for orientation analysis by applying a Confidence Index (CI) filter of 0.1 after performing a grain CI standardization. No further cleanups which actually modify orientations were applied. Cut planes for SEM analyses were prepared by polishing with abrasive slurries down to a diamond paste of 1 µm grain size. A final finish of 30 min using colloidal silica was applied. All samples were contacted with Ag-paste and coated with a thin layer of carbon at about 10^−3^ Pa to avoid surface charging in the SEM.

The piezoelectric constant d_33_ of the glass-ceramic D was studied using a d_33_-meter (Sinocera YE2730A). The extracted sample was coated with sputtered gold electrodes of ~150 nm thickness (VEB Hochvakuum Dresden B30) on the opposing sides.
